# Supplementary material for: Epidemic graph diagrams as analytics for epidemic control in the data-rich era
Source: Nat Commun. 2023 Dec 20;14:8472. doi: 10.1038/s41467-023-43856-1 (PMC10733371; doi:10.1038/s41467-023-43856-1)
Supplement: Supplementary file 1 — Supplementary Information [file 41467_2023_43856_MOESM1_ESM.pdf]

Supplementary Information

Epidemic graph diagrams as analytics for epidemic control in the data-rich era

Eugenio Valdano[1], Davide Colombi[2],
Chiara Poletto[1], and Vittoria Colizza[1].

[1]INSERM, Sorbonne Université, Institut Pierre Louis d'Epidémiologie et de Santé Publique, F75012, Paris, France.
[2]aizoOn Technology Consulting, 10146 Turin, Italy.

1 Syphilis

We illustrate here the compartmental model of syphilis introduced in [1] considering the full disease progression. Compared to the model we use in the main paper, it differentiates between primary and secondary stages, and includes later stages of the disease, such as latent syphilis, reactivation of secondary syphilis, tertiary syphilis. The classic representation of the compartmental model is shown in Figure S1. Once infected, susceptible individuals enter the exposed compartment (E) before developing primary syphilis infection (compartment I_1). If not treated, they then progress to secondary syphilis (I_2). After that, a long period of spontaneous remission follows (latent syphilis L). The last stage of untreated syphilis is then tertiary syphilis (I_3). Before ending in I_3 some individuals present recurrent episodes of secondary syphilis (I_R). The latent stage preceding I_R is in general shorter and is modelled with a different compartment (branch between L and L_R) in the figure.

Treatment may occur at any time during the disease progression. Upon treatment, individuals with primary and secondary infection are cured and become susceptible again, while individuals in compartments L_R, L, I_R and I_3 retain temporary immunity after cure. Transition parameters are indicated in Figure S1.

We assume that individuals with primary and secondary infection (I_1, I_2 and I_R) have the same transmission rate λ, and latent and tertiary syphilis are not infectious. However, the EGD could be easily modified to accommodate violations of these assumptions, which would simply change the value of the final effective parameters in Eqs. (S4) and (S5) here below.

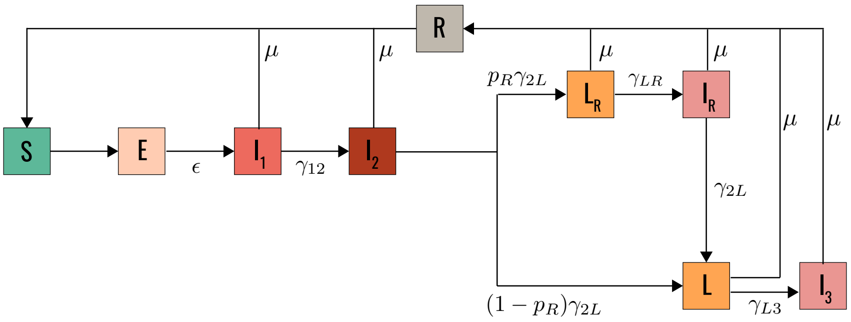

Figure S1: Compartmental model of syphilis considering the full natural history of the disease [1].

The EGD of the full compartmental model of syphilis is shown in Figure S2. We first `CUT` the diagram as shown by the two strongly connected components

highlighted in the figure. Compartments R, L, and I_3 can be `CUT` out leaving a remaining subdiagram of 5-nodes (top component, nodes: $\mathrm{E}, \mathrm{I}_1, \mathrm{I}_2, \mathrm{L}_R, \mathrm{I}_R$). If the weak-commutation condition holds, we `ZIP` this subdiagram and obtain an EGD of an SIS model whose effective parameters (see Methods of main paper) are

$$\mu_{eff} = \epsilon; \tag{S1}$$

$$\lambda_{eff} = -\epsilon \sum_s \lambda_s (J_X^{-1})_{1s}, \tag{S2}$$

where J_X, the Jacobian of the subdiagrams generated by nodes $\mathrm{I}_1, \mathrm{I}_2, \mathrm{L}_R, \mathrm{I}_R$, is given by

$$J_X = \begin{pmatrix} -\mu - \gamma_{12} & \gamma_{12} & 0 & 0 \\ 0 & -\mu - \gamma_{2L} & p_R\gamma_{2L} & 0 \\ 0 & 0 & -\mu - \gamma_{LR} & \gamma_{LR} \\ 0 & 0 & 0 & -\mu - \gamma_{2L} \end{pmatrix}. \tag{S3}$$

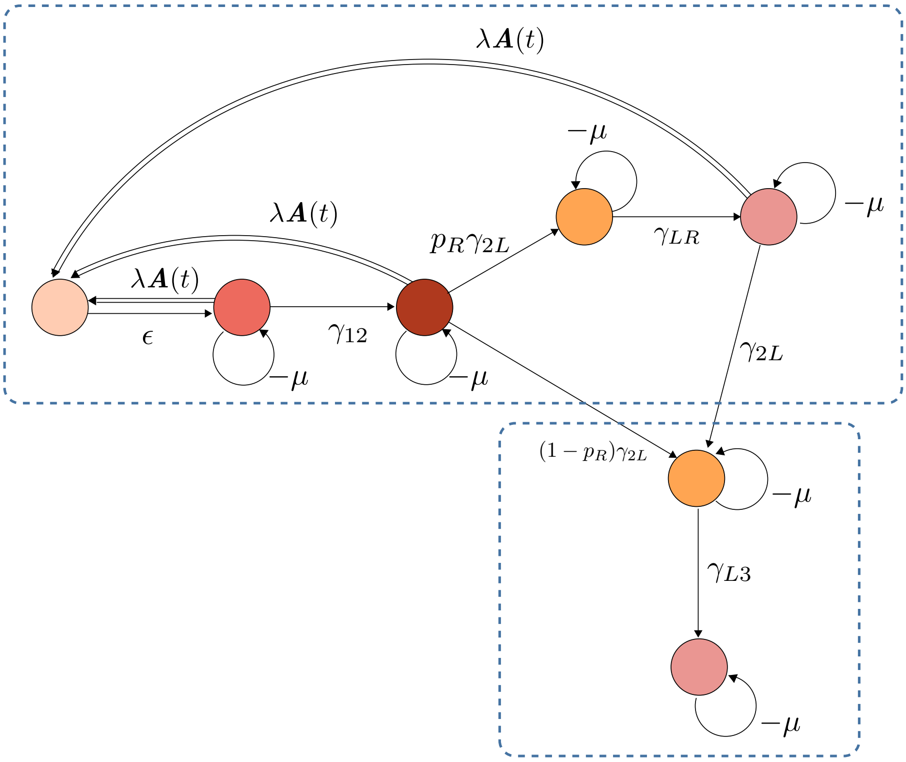

Figure S2: EGD of the syphilis model of Fig. S1.

It is convenient to use the epidemic threshold formula of the SIS model under weak-commutation condition to simplify the parameters further: λ_{eff}/μ_{eff} =

$1/\rho[\bar{A}]$. From this we can simplify both λ_{eff} to μ_{eff}, and remove ϵ: under weak-commutation the incubation period does not impact the threshold, as we showed in the main paper for the simpler SEIRS model. Using the scaling $\lambda_{eff}/\mu_{eff} = 1/\rho[\bar{A}]$, we can also redefine the effective parameters so that λ_{eff} matches syphilis transmissibility λ, and μ_{eff} contains the multistage corrections:

$$\lambda_{eff} = \lambda; \tag{S4}$$

$$\mu_{eff} = (\mu + \gamma_{12}) \left\{ 1 + \frac{\gamma_{12} \left[\mu(\mu + \gamma_{LR}) + \gamma_{2L}(\mu + (1 + p_R)\gamma_{LR}) \right]}{(\mu + \gamma_{LR})(\mu + \gamma_{2L})^2} \right\}^{-1} \tag{S5}$$

2 Influenza

We model influenza and antiviral treatment following the approach of Ref. [2] (Figure S3). We consider two antiviral drugs and four strains with different resistance profiles:

- wild-type (subscript w);
- mono-resistant: resistant to either drug (subscript 1 or 2);
- multi-resistant: resistant to both drugs (subscript 12).

Susceptible individuals (S) can get infected by one strain and enter the exposed compartment ($\mathrm{E_y}$ with $\mathrm{y} = \mathrm{w}, 1, 2, 12$). After an average latency period ϵ^{-1} individuals become pre-symptomatic ($\mathrm{P_y}$) – i.e. infectious, with no clinical manifestation. A proportion p_S of them develops symptoms with rate ϵ_P and enters the $\mathrm{I_y}$ compartment, while the rest remains asymptomatic ($\mathrm{A_y}$ compartment). Asymptomatic individuals have a shorter infectious duration compared to symptomatic individuals ($\mu_A^{-1} < \mu_I^{-1}$).

Upon onset of symptoms, treatment is administered with rate ν to a fraction of symptomatic, selected with probability p_T. Treated individuals who do not develop resistance (additional resistance if resistance to one drug is already present) go from $\mathrm{I_y}$ to $\mathrm{T_y}$. Those who do go from $\mathrm{I_y}$ to the treated infectious compartment corresponding to a different strain. As an example, the following outcome is possible for $\mathrm{I_w}$ individuals:

- they enter the compartment $\mathrm{T_w}$ (treated, infected with the wilde-type virus) with rate $\nu p_T(1-p_1)(1-p_2)$, where p_1 (p_2) is the probability of developing resistance to drug 1 (2);
- they enter the compartment $\mathrm{T_1^m}$ (or $\mathrm{T_2^m}$) with rate $\nu p_{\mathrm{T}} p_1(1-p_2)$ ($\nu p_{\mathrm{T}} p_2(1-p_1)$) corresponding to treated individuals infected with mutant virus of type 1 (2), if resistance is developed upon treatment;

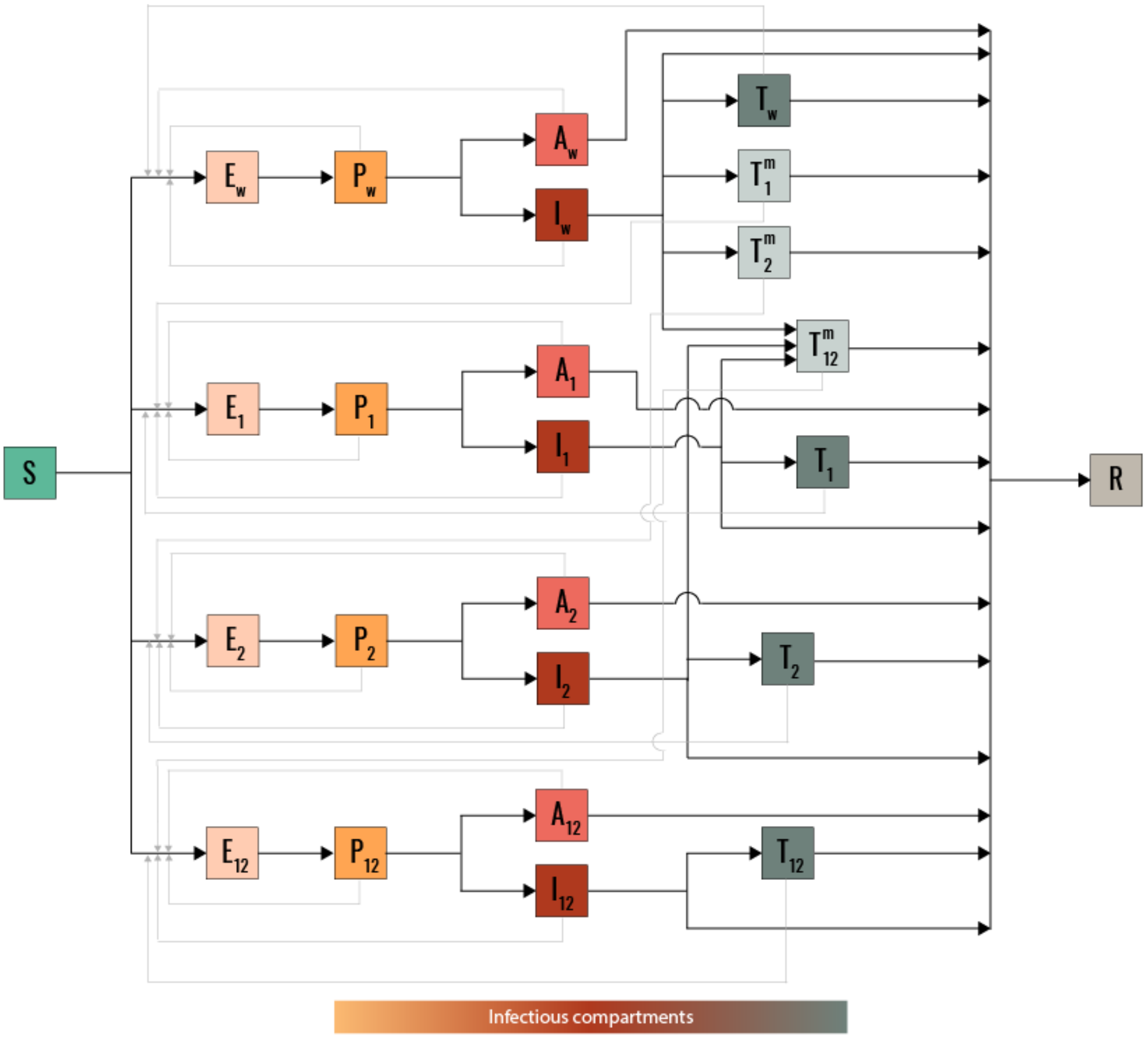

Figure S3: Compartmental model for pandemic influenza with antiviral combination therapy and development of drug resistance [2].

- they enter the compartment $\mathrm{T}^{\mathrm{m}}_{12}$ with rate $\nu p_{\mathrm{T}} p_1 p_2$ corresponding to treated individuals infected with the multi-resistant strain, if multi-resistance is acquired;

- they go untreated, i.e. transit directly from $\mathrm{I_w}$ to the recovered R compartment with rate $\mu_I(1 - p_T)$, where μ_I is the recovery rate of untreated infectious individuals.

Mutations from mono-resistant to the wild-type or from multi-resistant to mono-resistant are not considered in the model [2]. We assume treatment to reduce duration of infection, with τ being the difference between the overall infection duration in treated and untreated individuals, i.e. $\mu_I^{-1} = \nu^{-1} + \mu_T^{-1} + \tau$. Treatment is still effective for mono-resistant strains since we assume a combined therapy. However, it has no effect on multi-resistant strain. Thus the overall infectious duration for the latter is the same as untreated individuals, i.e. $\mu_I^{-1} = \nu^{-1} + \mu_{mm}^{-1}$.

Transmissibility depends on strain and degree of symptoms. Being λ the transmission rate of $\mathrm{I_w}$ taken as baseline value, transmission in the pre-symptomatic, asymptomatic and treated class is rescaled of a factor h_P, h_A and h_T, respectively. At the same time, mutation may alter the transmission potential of the virus. Mutant strains are often (but not always) less fit [2, 3], thus we introduce the fitness cost parameter ϕ_1 (ϕ_2), and we explore both positive and negative values, where negative fitness cost means increased transmissibility upon mutation.

Parameters and their values are summarised in Table S1. For the analysis of Fig. 4b-e of the main paper we parametrise the model as in [2, 4, 5] and vary the values of ϕ_1, ϕ_2 and p_T computing the corresponding threshold value of λ.

2.1 `CUT` of pandemic influenza EGD

At first, `CUT` separates the recovered compartment from the remaining part of the $\mathcal{EGD}$ – see Fig. 4a of main paper. The latter can be then further cut into 4 diagrams: $\mathcal{EGD}_w, \mathcal{EGD}_1, \mathcal{EGD}_2, \mathcal{EGD}_{12}$. The weights of the links are reported in Table S2 ($\mathcal{EGD}_w, \mathcal{EGD}_1, \mathcal{EGD}_2$), and Table S3 ($\mathcal{EGD}_{12}$).

2.2 `ZIP` of pandemic influenza EGD

All four submodels of the influenza model are `ZIP`pable. The effective parameters μ_{eff}, λ_{eff} of $\mathcal{EGD}_w, \mathcal{EGD}_1, \mathcal{EGD}_2, \mathcal{EGD}_{12}$ are

$$\mu_{eff}^{(x)} = \epsilon; \tag{S6}$$

$$\lambda_{eff}^{(x)} = -\epsilon\lambda(1 - \phi_x) \sum_j h_j \left[\left(J^{(x)} \right)^{-1} \right]_{1j}, \tag{S7}$$

name	type	description	value/notes
		transmission	
λ	transmission rate	$= \lambda(\mathrm{I_w})$, baseline	threshold value computed
h_P	correction for pre-sym	$= \lambda(\mathrm{P_x})/\lambda(\mathrm{I_x})$	0.68
h_A	correction for asym	$= \lambda(\mathrm{A_x})/\lambda(\mathrm{I_x})$	0.36
h_T	correction for treated	$= \lambda(\mathrm{T_x})/\lambda(\mathrm{I_x})$	0.67
ϕ_1	fitness cost	$= 1 - \lambda(\mathrm{I_1})/\lambda(\mathrm{I_w})$	explored
ϕ_2	fitness cost	$= 1 - \lambda(\mathrm{I_2})/\lambda(\mathrm{I_w})$	explored
		branching	
p_S	prob symptoms		0.67
p_T	prob treatment		explored
p_1	prob resistance to 1		$10^{-2}, 10^{-1}$
p_2	prob resistance to 2		$10^{-2}, 10^{-1}$
τ	less inf. per. due to treatment		$1\,day$
		spontaneous transitions	
ϵ	transition rate	E to P	$(0.98\,day)^{-1}$
ϵ_P	transition rate	P to A, I	$(0.5\,day)^{-1}$
ν	treatment rate		$(0.5\,day)^{-1}$
μ_I	recovery rate	for I	$(2.9\,day)^{-1}$
μ_A	recovery rate	for A	$(0.5\,day)^{-1}$
		derived variables	
μ_T	recovery rate	for T	from $\mu_I^{-1} = \nu^{-1} + \mu_T^{-1} + \tau$
μ_m	recovery rate	for $\mathrm{T_1^{(m)}}, \mathrm{T_2^{(m)}}$	$= \mu_T$
μ_{mm}	recovery rate	for $\mathrm{T_{12}^{(m)}}$	from $\mu_I^{-1} = \nu^{-1} + \mu_{mm}^{-1}$

Table S1: Parameters of the pandemic influenza model. $\lambda(C)$ is the transmissibility of compartment C. Subscript x takes values $w, 1, 2$. Parameter values from Ref. [2, 4, 5]

source	target	type	value
		$\mathcal{EGD}_w$	
E	P	s	ϵ
P	A	s	$\epsilon_P(1-p_S)$
P	I	s	$\epsilon_P p_S$
I	T	s	$\nu p_T(1-p_1)(1-p_2)$
A	A	s	$-\mu_A$
I	I	s	$-\mu_I - \nu p_T[1-(1-p_1)(1-p_2)]$
T	T	s	$-\mu_T$
P	E	d	$h_P \lambda A(t)$
I	E	d	$\lambda A(t)$
A	E	d	$h_A \lambda A(t)$
T	E	d	$h_T \lambda A(t)$
		$\mathcal{EGD}_1$	
E	P	s	ϵ
P	A	s	$\epsilon_P(1-p_S)$
P	I	s	$\epsilon_P p_S$
I	T	s	$\nu p_T(1-p_2)$
A	A	s	$-\mu_A$
I	I	s	$-\mu_I - \nu p_T p_2$
T	T	s	$-\mu_T$
P	E	d	$(1-\phi_1)h_P \lambda A(t)$
I	E	d	$(1-\phi_1)\lambda A(t)$
A	E	d	$(1-\phi_1)h_A \lambda A(t)$
T	E	d	$(1-\phi_1)h_T \lambda A(t)$
		$\mathcal{EGD}_2$	
E	P	s	ϵ
P	A	s	$\epsilon_P(1-p_S)$
P	I	s	$\epsilon_P p_S$
I	T	s	$\nu p_T(1-p_1)$
A	A	s	$-\mu_A$
I	I	s	$-\mu_I - \nu p_T p_1$
T	T	s	$-\mu_T$
P	E	d	$(1-\phi_2)h_P \lambda A(t)$
I	E	d	$(1-\phi_2)\lambda A(t)$
A	E	d	$(1-\phi_2)h_A \lambda A(t)$
T	E	d	$(1-\phi_2)h_T \lambda A(t)$

Table S2: Links of the subdiagrams $\mathcal{EGD}_w, \mathcal{EGD}_1, \mathcal{EGD}_2$, resulting from the `CUT`. Parameters are described in Table S1.

source	target	type	value
		$\mathcal{EGD}_{12}$	
E	P	s	ϵ
P	A	s	$\epsilon_P(1-p_S)$
P	I	s	$\epsilon_P p_S$
A	A	s	$-\mu_A$
I	I	s	$-\mu_I$
P	E	d	$(1-\phi_1)(1-\phi_2)h_P\lambda A(t)$
I	E	d	$(1-\phi_1)(1-\phi_2)\lambda A(t)$
A	E	d	$(1-\phi_1)(1-\phi_2)h_A\lambda A(t)$

Table S3: Links of the subdiagram $\mathcal{EGD}_{12}$, resulting from the `CUT`. Parameters are described in Table S1.

where x takes values $w, 1, 2, 12$; $J^{(x)}$ is the Jacobian of $\mathcal{EGD}_x - \{E\}$, ϕ_x is the fitness cost corresponding to $\mathcal{G}_x$ and $h = (h_P, h_A, 1, h_T)$.

These are the relevant Jacobian matrices:

$$J_w = \begin{pmatrix} -\epsilon_P & \epsilon_P(1-p_S) & \epsilon_P p_S & 0 \\ 0 & -\mu_A & 0 & 0 \\ 0 & 0 & -\mu_I - \nu p_T & \nu p_T(1-p_1)(1-p_2) \\ 0 & 0 & 0 & -\mu_T \end{pmatrix} \tag{S8}$$

$$J_1 = \begin{pmatrix} -\epsilon_P & \epsilon_P(1-p_S) & \epsilon_P p_S & 0 \\ 0 & -\mu_A & 0 & 0 \\ 0 & 0 & -\mu_I - \nu p_T & \nu p_T(1-p_2) \\ 0 & 0 & 0 & -\mu_T \end{pmatrix} \tag{S9}$$

$$J_2 = \begin{pmatrix} -\epsilon_P & \epsilon_P(1-p_S) & \epsilon_P p_S & 0 \\ 0 & -\mu_A & 0 & 0 \\ 0 & 0 & -\mu_I - \nu p_T & \nu p_T(1-p_1) \\ 0 & 0 & 0 & -\mu_T \end{pmatrix} \tag{S10}$$

$$J_{12} = \begin{pmatrix} -\epsilon_P & \epsilon_P(1-p_S) & \epsilon_P p_S \\ 0 & -\mu_A & 0 \\ 0 & 0 & -\mu_I \end{pmatrix}. \tag{S11}$$

And the first row of their inverses is

$$\begin{aligned}
\left[\left(J^{(w)}\right)^{-1}\right]_{1j} &= -\left(\epsilon_P^{-1}, (1-p_S)\mu_A^{-1}, p_S\left(\mu_I+\nu p_T\right)^{-1},\right. \\
&\qquad \left. p_S p_T \nu (1-p_1)(1-p_2)\left(\mu_I+\nu p_T\right)^{-1}\mu_T^{-1}\right); \\
\left[\left(J^{(1)}\right)^{-1}\right]_{1j} &= -\left(\epsilon_P^{-1}, (1-p_S)\mu_A^{-1}, p_S\left(\mu_I+\nu p_T\right)^{-1},\right. \\
&\qquad \left. p_S p_T \nu (1-p_2)\left(\mu_I+\nu p_T\right)^{-1}\mu_T^{-1}\right); \\
\left[\left(J^{(2)}\right)^{-1}\right]_{1j} &= -\left(\epsilon_P^{-1}, (1-p_S)\mu_A^{-1}, p_S\left(\mu_I+\nu p_T\right)^{-1},\right. \\
&\qquad \left. p_S p_T \nu (1-p_1)\left(\mu_I+\nu p_T\right)^{-1}\mu_T^{-1}\right); \\
\left[\left(J^{(12)}\right)^{-1}\right]_{1j} &= -\left(\epsilon_P^{-1}, (1-p_S)\mu_A^{-1}, p_S\mu_I^{-1}, 0\right). \qquad \text{(S12)}
\end{aligned}$$

The vector $\left[\left(J^{(12)}\right)^{-1}\right]_{1j}$ is actually 3-dimensional. We have added a zero fourth entry to so that the general scalar product in Eq. (S7) holds.

$$\omega = h_P/\epsilon_P + (1-p_S)h_A/\mu_A, \qquad \text{(S13)}$$

$$\sigma = \frac{p_S}{\mu_I}, \qquad \text{(S14)}$$

$$\hat{\nu} = \nu p_T \ \ \text{(they always appear together)}, \qquad \text{(S15)}$$

$$\xi(\hat{\nu}) = \left(1+\frac{\hat{\nu}}{\mu_I}\right)^{-1}, \qquad \text{(S16)}$$

$$\psi = \frac{h_T}{\mu_T}. \qquad \text{(S17)}$$

Using the values in Table S1, we get $\omega = 9.59\,hour$, $\sigma = 46.63\,hour$, $\psi = 22.51\,hour$. We assume $p_1 = p_2 = p$, and write the `ZIP` equations for the effective SIS parameters at the critical point (Eq. (S7)):

$$\left\{\lambda^{(w)}_{critical}\,\rho[\bar{A}]\right\}^{-1} = \omega + \sigma\xi(\hat{\nu})\left[1+\psi(1-p)^2\hat{\nu}\right]; \qquad \text{(S18)}$$

$$\left\{\lambda^{(1)}_{critical}\,\rho[\bar{A}]\right\}^{-1} = (1-\phi_1)\left\{\omega + \sigma\xi(\hat{\nu})\left[1+\psi(1-p)\hat{\nu}\right]\right\}; \qquad \text{(S19)}$$

$$\left\{\lambda^{(12)}_{critical}\,\rho[\bar{A}]\right\}^{-1} = (1-\phi_1)(1-\phi_2)\left(\omega+\sigma\right). \qquad \text{(S20)}$$

From finding the minimum among Eq. (S18,S19,S20), we recover the parameter surfaces that separate the different dominance phases (Fig. 4d of main paper). The network component $\rho[\bar{A}]$ always factors, so that network topology does not influence dominance [6] if the weak-commutation condition holds. For these reasons the following quantities are valid for any cntact network:

$$\hat{\nu}_{w-12} = \mu_I \frac{(\omega+\sigma)(\phi_1+\phi_2-\phi_1\phi_2)}{(1-\phi_1)(1-\phi_2)\sigma - \omega(\phi_1+\phi_2-\phi_1\phi_2) - p_S\frac{h_T}{\mu_T}(1-p)^2}; \quad \text{(S21)}$$

$$\hat{\nu}_{w-1} = \frac{(\omega+\sigma)(1-\phi_1)}{\sigma\psi(1-p)(p-\phi_1) - \frac{\omega}{\mu_I}\phi_1}; \quad \text{(S22)}$$

$$\hat{\nu}_{1-12} = \frac{-\phi_2(\omega+\sigma)}{\sigma\psi(1-p) + \frac{\omega-(1-\phi_2)(\omega+\sigma)}{\mu_I}}. \quad \text{(S23)}$$

We also define

$$g = \sigma\frac{1-\mu_I(1-p)^2\psi}{\omega+\sigma}; \quad \text{(S24)}$$

$$\hat{g} = \frac{\psi p(1-p)}{\psi(1-p)+\omega/p_S}; \quad \text{(S25)}$$

$$\tilde{g} = \sigma\frac{1-\mu_I(1-p)\psi}{\omega+\sigma}. \quad \text{(S26)}$$

We then have that if $\phi_1+\phi_2-\phi_1\phi_2 > g$, then always $\lambda^{(w)}_{critical} < \lambda^{(12)}_{critical}$. If $0 < \phi_1+\phi_2-\phi_1\phi_2 < g$, then $\lambda^{(w)}_{critical} < \lambda^{(12)}_{critical} \Leftrightarrow \hat{\nu} < \hat{\nu}_{w-12}$, and $\lambda^{(w)}_{critical} > \lambda^{(12)}_{critical} \Leftrightarrow \hat{\nu} > \hat{\nu}_{w-12}$. Finally, if $\phi_1+\phi_2-\phi_1\phi_2 < 0$, then always $\lambda^{(w)}_{critical} > \lambda^{(12)}_{critical}$.

We then have that if $\phi_1 > \hat{g}$, then always $\lambda^{(w)}_{critical} < \lambda^{(1)}_{critical}$. If $0 < \phi_1 < \hat{g}$, then $\lambda^{(w)}_{critical} < \lambda^{(1)}_{critical} \Leftrightarrow \hat{\nu} < \hat{\nu}_{w-1}$, and $\lambda^{(w)}_{critical} > \lambda^{(1)}_{critical} \Leftrightarrow \hat{\nu} > \hat{\nu}_{w-1}$. Finally, if $\phi_1 < 0$, then always $\lambda^{(w)}_{critical} > \lambda^{(1)}_{critical}$.

We then have that if $\phi_2 > \tilde{g}$, then always $\lambda^{(1)}_{critical} < \lambda^{(12)}_{critical}$. If $0 < \phi_2 < \tilde{g}$, then $\lambda^{(1)}_{critical} < \lambda^{(12)}_{critical} \Leftrightarrow \hat{\nu} < \hat{\nu}_{1-12}$, and $\lambda^{(1)}_{critical} > \lambda^{(12)}_{critical} \Leftrightarrow \hat{\nu} > \hat{\nu}_{1-12}$. Finally, if $\phi_2 < 0$, then always $\lambda^{(1)}_{critical} > \lambda^{(12)}_{critical}$.

We can rewrite the last if we fix $\phi = \phi_1+\phi_2-\phi_1\phi_2$, and express as a function of ϕ_1. We then have that if $\phi_1 < (\phi-\tilde{g})/(1-\tilde{g})$, then always $\lambda^{(1)}_{critical} < \lambda^{(12)}_{critical}$. If $(\phi-\tilde{g})/(1-\tilde{g}) < \phi_1 < \phi$, then $\lambda^{(1)}_{critical} < \lambda^{(12)}_{critical} \Leftrightarrow \hat{\nu} < \hat{\nu}_{1-12}$, and $\lambda^{(1)}_{critical} > \lambda^{(12)}_{critical} \Leftrightarrow \hat{\nu} > \hat{\nu}_{1-12}$. Finally, if $\phi_1 > \phi$, then always $\lambda^{(1)}_{critical} > \lambda^{(12)}_{critical}$.

The values of $g, \hat{g}, \tilde{g}$ are in Table S4, which shows that typically $\hat{g}$ is small. This means that is difficult – but not impossible – to get to the dominance of the one-resistance form if neither one-resistant strain has an advantage in terms of transmission.

	g	$\hat{g}$	$\tilde{g}$
$p = 10^{-1}$	0.6122	0.0586	0.5880
$p = 10^{-2}$	0.5665	0.0061	0.5639
$p = 10^{-3}$	0.5617	0.0006	0.5615

Table S4: Numerical values of $g, \hat{g}, \tilde{g}$, defined in Eq. (S24,S25,S26), and calculated with the parameters in Table S1.

2.3 Additional parameters definition

Table S5 reports the definition of the additional parameters used in the rates of Fig. 4a of the main text. They are combinations of the parameters of the original model (Table S1).

parameter	value	index value
$\alpha_x =$	$(1-p_1)(1-p_2)$	for $x = w$
$=$	$(1-p_2)$	$x = 1$
$=$	$(1-p_1)$	$x = 2$
$\phi_x =$	0	for $x = w$
$=$	ϕ_1	$x = 1$
$=$	ϕ_2	$x = 2$
$\bar{p}_x =$	$1-(1-p_1)(1-p_2)$	for $x = w$
$=$	p_2	$x = 1$
$=$	p_1	$x = 2$
$\phi_y =$	ϕ_x	for $y = x$
$=$	$1-(1-\phi_1)(1-\phi_2)$	$y = 12$

Table S5: Definitions of the additional parameters used in Fig. 4a of the main text. The indices take the following values: $x = w, 1, 2$; $y = w, 1, 2, 12$, as in Fig 4a.

3 COVID-19

We model COVID-19 following Refs. [7, 8], using a notation similar to the one adopted to introduce the pandemic influenza case study. Once infected, susceptible individuals (S) enter the exposed compartment (E). After an average latency period ϵ^{-1} individuals become pre-symptomatic (P). A proportion $1 - p_S$ of them does not develop symptoms and enters compartment A at rate ϵ_P. The other become paucisymptomatic (I_{ps}, prob p_{ps}), mildly symptomatic (I_{ms}, prob p_{ms}), or develop severe symptoms (I_{ss}, $1 - p_{ms} - p_{ss}$). $\text{A}, \text{I}_{ps}, \text{I}_{ms}, \text{I}_{ss}$ recover at rate μ. I_{ss}

transition at the same rate to either hospitalization in intensive-care unit, ICU (U, prob p_{ICU}), or hospitalization outside ICU (H, prob $1 - p_{ICU}$). Both can either die or recover at specific rates. The compartmental scheme is shown in Figure S4.

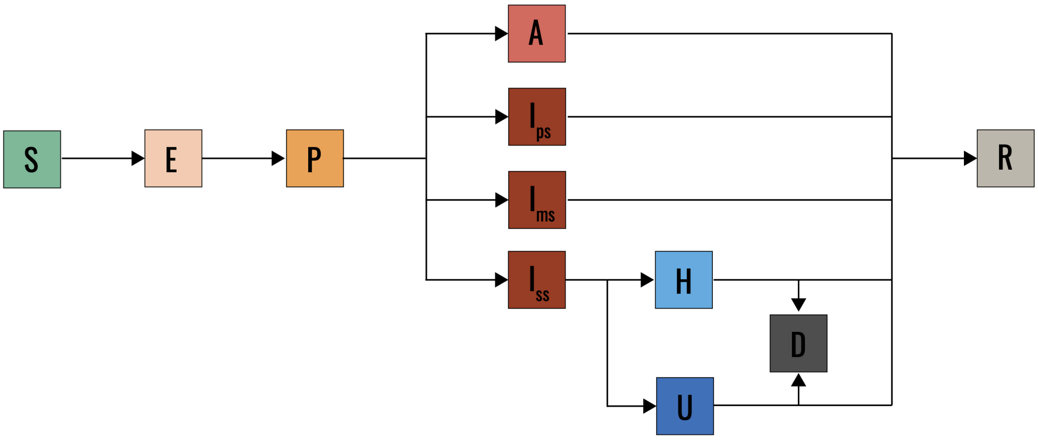

Figure S4: Compartmental model for COVID-19 [7,8].

Compartments $\mathrm{P}, \mathrm{A}, \mathrm{I}_{ps}, \mathrm{I}_{ms}, \mathrm{I}_{ss}$ can transmit SARS-CoV-2. Upon contact, $\mathrm{P}, \mathrm{A}, \mathrm{I}_{ps}$ do so at rate λ, while $\mathrm{I}_{ps}, \mathrm{I}_{ms}$ at rate $\eta\lambda$, with $\eta = 0.51$ as in Refs. [7,8].

A key aspect of this model is that transition probability depend on age. We consider four age classes (0-10; 11-18; 19-64; and 65+ years old, corresponding to children, adolescents, adults, and seniors), and the corresponding parameters are reported in Refs. [7,8]. Crucially, for instance, young individuals who develop symptoms are always paucisymptomatic ($p_{ps} = 1$), while $p_{ps} = 0.2$ for adults and seniors.

Practically this means parameters p_{ps}, p_{ms} are $N \times N$ diagonal matrices where ith diagonal entry contains the value corresponding to the age class of the ith individual.

As for the influenza model, the COVID-19 model can be `CUT`, and `ZIP`ed if in timescale separation.

Figure S5 reports the model after `CUT`. The `ZIP`ing process is similar to influenza's, with the difference that some parameters are matrices. This leads to the following equation of the threshold:

$$\lambda_c = \frac{1}{\rho\left[\bar{A}\left\{\epsilon_p^{-1} + \mu^{-1}(1 - p_S) + \mu^{-1} p_S\left[\eta + (1-\eta) p_{ps}\right]\right\}\right]}. \qquad \text{(S27)}$$

Below are the details of the `ZIP`ping.

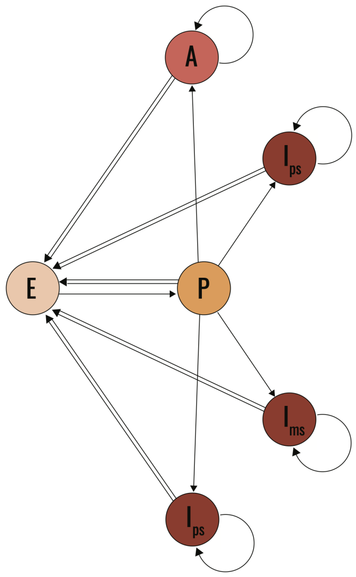

Figure S5: EGD of the COVID-19 model after CUT.

3.1 ZIP of COVID-19

The Jacobian of the relevant subdiagram reads

$$J_X = \begin{pmatrix} -\epsilon_P & \epsilon_P(1-p_S) & \epsilon_P p_S p_{ps} & \epsilon_P p_S p_{ms} & \epsilon_P p_S p_{ss} \\ 0 & -\mu & 0 & 0 & 0 \\ 0 & 0 & -\mu & 0 & 0 \\ 0 & 0 & 0 & -\mu & 0 \\ 0 & 0 & 0 & 0 & -\mu \end{pmatrix} . \quad \text{(S28)}$$

This can be inverted using block matrix inversion:

$$J_X^{-1} = \left(\begin{array}{c|c} -\epsilon_P^{-1} & -\mu^{-1}B \\ 0 & \mu^{-1} \end{array} \right) , \quad \text{(S29)}$$

with

$$B = \left[\begin{array}{cccc} 1-p_S & p_S p_{ps} & p_S p_{ms} & p_S(1-p_{ps}-p_{ms}) \end{array} \right] . \quad \text{(S30)}$$

From this, and by using ZIP equations, one gets to Eq. S27.

4 Discretization of the infection propagator

Often in the case of empirical data computing the infection propagator means discretizing Eq. (3) at the time resolution of the available data. We describe the discretization here. Straightforward discretization from Eq. (3) (or equivalently Eq. (5)) is possible, but cumbersome. It is instead easier, and more informative, to go back to Eq. (2) and discretize P from the beginning.

We first remark that P solves Eq. (2) of the main paper:

$$\dot{P}(t) = P(t)\left[\gamma - \mu + \lambda A(t)\right]. \tag{S31}$$

Then, we generalize the discretization found in [9]. Considering a discretization interval Δt, we can discretize this equation:

$$P(t + \Delta t) = P(t)\left[1 + \gamma\Delta t - \mu\Delta t + \lambda\Delta t A(t)\right]. \tag{S32}$$

This holds if Δt is small compared to the timescale of the system, as confirmed by the fact that Δt correctly transforms rates (γ, μ, λ) into probabilities $(p_\gamma = \gamma\Delta t, p_\mu = \mu\Delta t, p_\lambda = \lambda\Delta t)$ only when $p_\gamma, p_\mu, p_\lambda \ll 1$. Analogously, Eq. (S32) assumes that $A(t)$ is almost constant in intervals of length Δt. In practice, these mathematical requirements simply mean that the time resolution of the contact data should be appropriate to the epidemic process under study. We can now turn to a discrete notation:

$$P_{t+1} = P_t\left[1 + p_\gamma - p_\mu + p_\lambda A_{t+1}\right], \tag{S33}$$

where we have also used $\Delta t A(t) = \Delta t A(t + \Delta t)$, which is true at $\mathcal{O}(\Delta t)$.

The solution of Eq. (S33) (with usual initial condition $P_1 = 1$) is

$$P_t = \left[1 + p_\gamma - p_\mu + p_\lambda A_1\right]\left[1 + p_\gamma - p_\mu + p_\lambda A_2\right]\cdots\left[1 + p_\gamma - p_\mu + p_\lambda A_t\right]. \tag{S34}$$

5 Numerical validation

We tested the accuracy of the computation of the threshold from EGDs. We chose the popular and widely used activity-driven model [10] of a temporal network with the following parameters (in the notation of Ref. [10]): $N = 1000$, $T = 50$, $\gamma = 2.8$, $\epsilon = 0.01$, $\eta = 1$, $m = 7$. We used a SEIS compartmental model with per-contact transmission probability λ, E-to-I transition probability $\epsilon = 0.02$, recovery probability $\mu = \{0.05, 0.2\}$.

We then simulated the epidemic spread on this temporal network using both a stochastic microscopic numerical simulation and the numerical solution of the

microscopic Markov Chain equation (Eq. 1 in the manuscript). For both, we computed and plotted the value of the prevalence at the endemic state: the result is in Fig. S6, gray dots for the stochastic simulation, crosses for the Markov Chain equation. To compute the endemic state in the stochastic simulation we used the quasistationary state method as it was adapted to temporal networks in Ref. Valdano2015b. The figure plots the endemic prevalence at varying transmissibility λ and the epidemic threshold estimated with the epidemic graph diagrams (arrows). The threshold correctly predicts the critical transmissibility above which the prevalence is larger than zero.

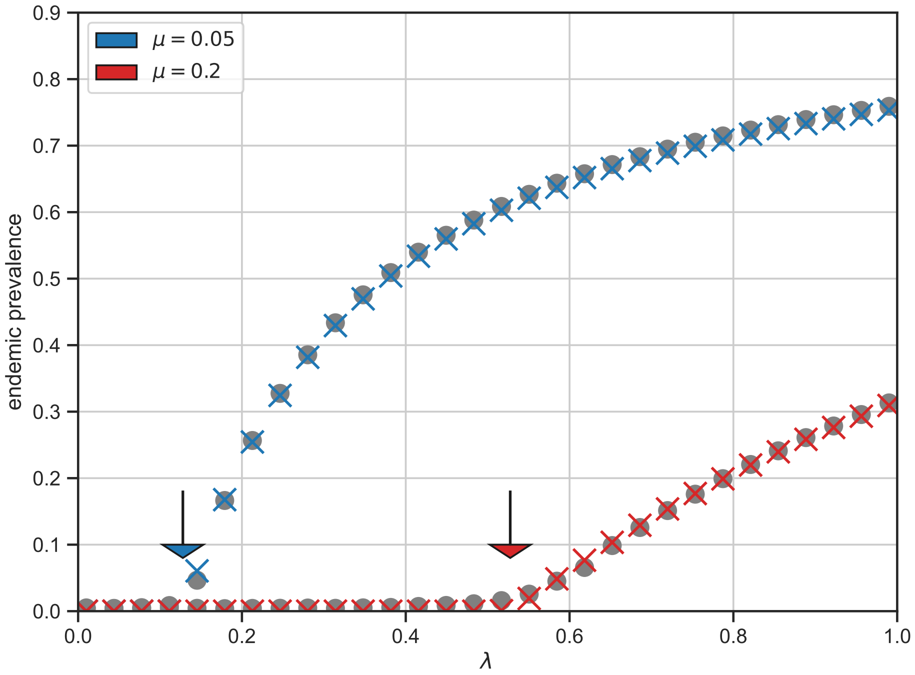

Figure S6: Validation of the threshold computation against numerical simulations. We simulated the spread of a an epidemic following the SEIS compartmental model on an instance of the activity-driven model of a temporal network. To generate the network we set the following parameters: $N = 1000$, $T = 50$, $\gamma = 2.8$, $\epsilon = 0.01$, $\eta = 1$, $m = 7$, in the notation of Ref. [10]. To simulate the epidemic we used a stochastic microscopic numerical simulation and the numerical solution of the microscopic Markov Chain equation (Eq. 1 in the paper). For both, we computed and plotted the value of the prevalence at the endemic state (gray dots for the stochastic simulation, crosses for the Markov Chain equation), at varying transmissibility λ. To compute the endemic state in the stochastic simulation we used the quasistationary state method as it was adapted to temporal networks [11]. The arrows indicate the epidemic threshold from epidemic graph diagrams. The fixed parameters of the SEIS compartmental model are the E-to-I transition probability $\epsilon = 0.02$ and the recovery probability $\mu = \{0.05, 0.2\}$.

Supplementary References

[1] Geoff Garnett, Sevgi Aral, Deborah Hoyle, Willard Cates, and Roy Anderson. The Natural History of Syphilis: Implications for the Transmission Dynamics and Control of Infection. Sexually Transmitted Diseases, 24(4):185–200, April 1997.

[2] Joseph T. Wu, Gabriel M. Leung, Marc Lipsitch, Ben S. Cooper, and Steven Riley. Hedging against Antiviral Resistance during the Next Influenza Pandemic Using Small Stockpiles of an Alternative Chemotherapy. PLOS Medicine, 6(5):e1000085, May 2009.

[3] Kathy Leung, Marc Lipsitch, Kwok Yung Yuen, and Joseph T Wu. Monitoring the fitness of antiviral-resistant influenza strains during an epidemic: A mathematical modeling study. The Lancet. Infectious diseases, 17(3):339–347, March 2017.

[4] Vittoria Colizza, Alain Barrat, Marc Barthélemy, Alain-Jacques Valleron, and Alessandro Vespignani. Modeling the Worldwide Spread of Pandemic Influenza: Baseline Case and Containment Interventions. PLoS Med, 4(1):e13, 2007.

[5] Matthew Biggerstaff, Simon Cauchemez, Carrie Reed, Manoj Gambhir, and Lyn Finelli. Estimates of the reproduction number for seasonal, pandemic, and zoonotic influenza: a systematic review of the literature. BMC infectious diseases, 14:480, September 2014.

[6] B. Aditya Prakash, Alex Beutel, Roni Rosenfeld, and Christos Faloutsos. Winner Takes All: Competing Viruses or Ideas on Fair-play Networks. In Proceedings of the 21st International Conference on World Wide Web, WWW '12, pages 1037–1046, New York, NY, USA, 2012. ACM. event-place: Lyon, France.

[7] Laura Di Domenico, Giulia Pullano, Chiara E. Sabbatini, Pierre-Yves Boëlle, and Vittoria Colizza. Impact of lockdown on COVID-19 epidemic in île-de-France and possible exit strategies. BMC Medicine, 18(1):240, July 2020.

[8] Giulia Pullano, Laura Di Domenico, Chiara E. Sabbatini, Eugenio Valdano, ClÃ©ment Turbelin, Marion Debin, Caroline Guerrisi, Charly Kengne-Kuetche, CÃ©cile Souty, Thomas Hanslik, Thierry Blanchon, Pierre-Yves BoÃ«lle, Julie Figoni, Sophie Vaux, Christine CampÃ¨se, Sibylle Bernard-Stoecklin, and Vittoria Colizza. Underdetection of cases of COVID-19 in

France threatens epidemic control. Nature, 590(7844):134–139, February 2021. Number: 7844 Publisher: Nature Publishing Group.

[9] Eugenio Valdano, Michele Re Fiorentin, Chiara Poletto, and Vittoria Colizza. Epidemic Threshold in Continuous-Time Evolving Networks. Physical Review Letters, 120(6):068302, 2018.

[10] Nicola Perra, Bruno Gonçalves, Romualdo Pastor-Satorras, and Alessandro Vespignani. Activity driven modeling of time varying networks. Sci. Rep., 2, jun 2012.

[11] Eugenio Valdano, Luca Ferreri, Chiara Poletto, and Vittoria Colizza. Analytical Computation of the Epidemic Threshold on Temporal Networks. Phys. Rev. X, 5(2):21005, apr 2015.
